# Correction: Bourahmah et al. Error Function Optimization to Compare Neural Activity and Train Blended Rhythmic Networks. *Brain Sci.* 2024, *14*, 468

**DOI:** 10.3390/brainsci14111044

**Published:** 2024-10-22

**Authors:** Jassem Bourahmah, Akira Sakurai, Paul S. Katz, Andrey L. Shilnikov

**Affiliations:** 1 Neuroscience Institute, Georgia State University, 100 Piedmont Ave., Atlanta, GA 30303, USA; bour8727@gmail.com (J.B.); akira@gsu.edu (A.S.); 2 Neuroscience & Behavior Graduate Program, Department of Biology, University of Massachusetts, Amherst, MA 01003, USA; pkatz@umass.edu; 3 Department of Mathematics & Statistics, Neuroscience Institute, Georgia State University, 100 Piedmont Ave., Atlanta, GA 30303, USA

## Addition of an Author

Paul S. Katz was not included as an author in the original publication [1]. The corrected Author Contributions statement appears here:

**Author Contributions:** Conceptualization, A.L.S.; Methodology, J.B., A.S. and A.L.S.; Software, A.S.; Validation, J.B. and A.S.; Investigation, J.B., A.S. and A.L.S.; Data curation, A.S. and P.S.K.; Writing—original draft, J.B. and A.L.S.; Visualization, J.B.; Supervision, A.L.S.; Funding acquisition, A.L.S. and P.S.K. All authors have read and agreed to the published version of the manuscript.

## Additional Affiliation

Affiliation 2 was newly added for Paul S. Katz. The updated affiliation should include the following: 2. Neuroscience & Behavior Graduate Program, Department of Biology, University of Massachusetts, Amherst, MA 01003, USA. The author Akira Sakurai’s affiliation has been changed to affiliation 1. The author Jassem Bourahmah’s email has been changed to bour8727@gmail.com.

## Funding Update

Updated Funding as below:

**Funding:** We thank the Brains & Behavior Initiative at Georgia State University for their support through the Brains & Behavior pilot grant, as well as for the B&B graduate fellowship awarded to J. Bourahmah. The authors thank the current and former members of the Shilnikov NeurDS (Neuro-Dynamical Systems) lab: J. Scully, D. Bloom, C. Hinsley, Y. Keleta, H. Ju, D. Alacam, and K. Pusuluri. This paper was partially funded by the National Science Foundation award, no. IOS-1455527.

## Text Correction

There was an error in the original publication [1]. No methods for the electrophysiology were included in the original manuscript. A correction has been made to Section 2. Methods. At the beginning of Section 2.1, a new paragraph was added.

Experimental methodology: The methods for the electrophysiological experiments are the same as those reported in Ref. [46].

## Reference

Ref. [46] was added as follows:

46. Sakurai, A.; Katz, P.S. Command or Obey? Homologous neurons differ in hierarchical position for the generation of homologous behaviors. *J. Neurosci.* **2019**, *39*, 6460–6471. https://doi.org/10.1523/JNEUROSCI.3229-18.2019.

With this correction, the order of some of the references has been adjusted accordingly. The authors state that the scientific conclusions are unaffected. This correction was approved by the Academic Editor. The original publication has also been updated.
